# Development of novel aptamers for low-density lipoprotein particle quantification

**DOI:** 10.1371/journal.pone.0205460

**Published:** 2018-10-11

**Authors:** Daniel Klapak, Sarah Broadfoot, Gregory Penner, Anup Singh, Eshwar Inapuri

**Affiliations:** 1 NeoVentures Biotechnology Inc., London, Ontario, Canada; 2 InnaMed, Inc., Philadelphia, Pennsylvania, United States of America; Consiglio Nazionale delle Ricerche, ITALY

## Abstract

Cardiovascular disease (CVD) remains the leading cause of death worldwide. Low-density lipoprotein cholesterol (LDL-C) is commonly used for CVD risk assessment; however, recent research has shown LDL particle (LDL-P) number to be a more sensitive indicator of CVD risk than both LDL-C and non-high-density lipoprotein cholesterol (HDL-C). Described herein are five single stranded DNA aptamers with dissociation constants in the low picomolar range specific to LDL-P and its subfractions. Furthermore, a set of antisense sequences have been developed and characterized that are capable of binding to the best aptamers and undergoing displacement by LDL-P for use in a simple, affordable diagnostic assay.

## Introduction

Cardiovascular disease (CVD) remains the leading cause of death worldwide [1]. Currently, a lipid panel test, consisting of total cholesterol, high-density lipoprotein cholesterol (HDL-C), low-density lipoprotein cholesterol (LDL-C) and triglycerides is the standard screening method for CVD risk with a high LDL-C and a low HDL-C level indicating an increased risk of CVD. However, recent research has shown that in a significant portion of the population there is a discordance between LDL-C and LDL particle count (LDL-P) [2]. A high LDL-P is associated with a higher risk of CVD due to the favorable diffusive and aggregative interaction of the particles with vascular walls. For those with this discordance, only LDL-P was associated with incident CVD [3]. Moreover, several studies suggest LDL-P and its small and dense particle subfraction are superior biomarkers to LDL-C and non-HDL-C for CVD risk assessment and screening [4, 5]. This discordance between LDL-C and LDL-P frequently goes unnoticed in traditional lipid testing. As a result, a sizeable portion of the population at risk for CVD are not identified and do not receive the necessary therapy to reduce risk of disease. Conversely, a sizable portion of the population receive therapies when they may not be needed and could potentially be harmful [6].

Currently, the main methods for measuring LDL-P are nuclear magnetic resonance [7], ultracentrifugation [8] and gas-phase electrophoresis [9]. These techniques require bulky machinery and specialized technicians making them expensive and time-consuming to carry out. Additionally, this advanced lipoprotein testing requires a separate blood sample to be collected and sent to a reference lab for analysis. Due to high cost, time and difficulty, LDL-P tests are rarely performed despite the National Lipid Association advocating for its increased utilization in their guidelines [10].

Aptamers are short oligonucleotides that bind to small molecules, proteins and even tissues/cells with high affinity and specificity [11]. Aptamers are traditionally isolated using a process called Systematic Evolution of Ligands by EXponential enrichment (SELEX) that was discovered independently by the Gold and Szostak Labs in 1990 [12] [13]. The various SELEX methodologies follow the same general process wherein a large set (e.g. 10^15^) of random oligonucleotide sequences is subjected to repeated selection rounds (via exposure to the immobilized target of interest) and amplification. The final sequences obtained from this process with the highest specificity and affinity to the target of interest are then amplified for further study and use.

Here, a free selection strategy (FRELEX) was employed that, unlike the SELEX process, does not require the immobilization of the target or the oligonucleotide library [14]. Immobilization of target molecules has the potential to constrain the expression of important epitopes that could be exposed in free form and involved in oligonucleotide binding interactions.

Despite the widespread use of antibodies, aptamers have high potential as diagnostic tools. The primary advantage of using aptamers over antibodies is their smaller size. A smaller size improves access to an increased number of epitopes on target molecules, and enables higher rates of diffusion in solution for faster measurements with minimal batch-to-batch variation due to high fidelity chemical synthesis [15]. This ability for rapid *in vitro* synthesis also reduces production costs. Additionally, aptamers can be chemically modified to greatly increase stability and can easily be incorporated in simple, sensitive assay formats [16]. These unique properties make aptamers flexible and powerful tools for applications in diagnostics.

Single-stranded DNA (ssDNA) aptamers have been selected for a wide variety of targets including small molecules (e.g. cocaine, ATP, cholic acid) and proteins (e.g. thrombin, IgE, troponin) [17]. Aptamers have been used in biosensors for target detection in body fluids, food samples, and other complex matrices. The biosensors are designed so that the binding of the aptamer to the target of interest elicits a change in electrochemical signal, optical signal or mass of the substance [18]. To date, no published aptamers have been selected for LDL particles or Apolipoprotein B100 (ApoB100), the largest of the ApoB group of proteins on the LDL particle surface. Also, no size- or density-based aptamer selection has been published for LDL particles.

The present work describes the selection of ssDNA aptamers against LDL particles using the FRELEX process. By leveraging the versatility of aptamers, ligands that bind LDL particles can be successfully developed where previously they have not been developed using antibodies. In this work, five aptamers were identified that bind to LDL particles with extremely high affinities. The aptamer LDL-A1 appeared to bind with higher affinity to large and fluffy LDL (LF-LDL) than to small and dense LDL (SD-LDL). After further binding studies in dilute blood serum samples and in HDL and human serum albumin solutions, LDL-A1 appeared to be the best aptamer for LDL particles with a 1.6 pM dissociation constant (k_D_) and minimal binding to HDL and HSA. To our knowledge, this is the first set of aptamers developed that bind to LDL particles selectively and can be used to differentiate between SD-LDL and LF-LDL. To apply these aptamers in a simple assay format, antisense sequences were designed for LDL-A1, A2 and A12 that will bind to the respective aptamers, but from which the aptamer is still displaceable via the introduction of LDL-P.

## Materials and methods

### Isolation of LDL particles

**Fig 1:**
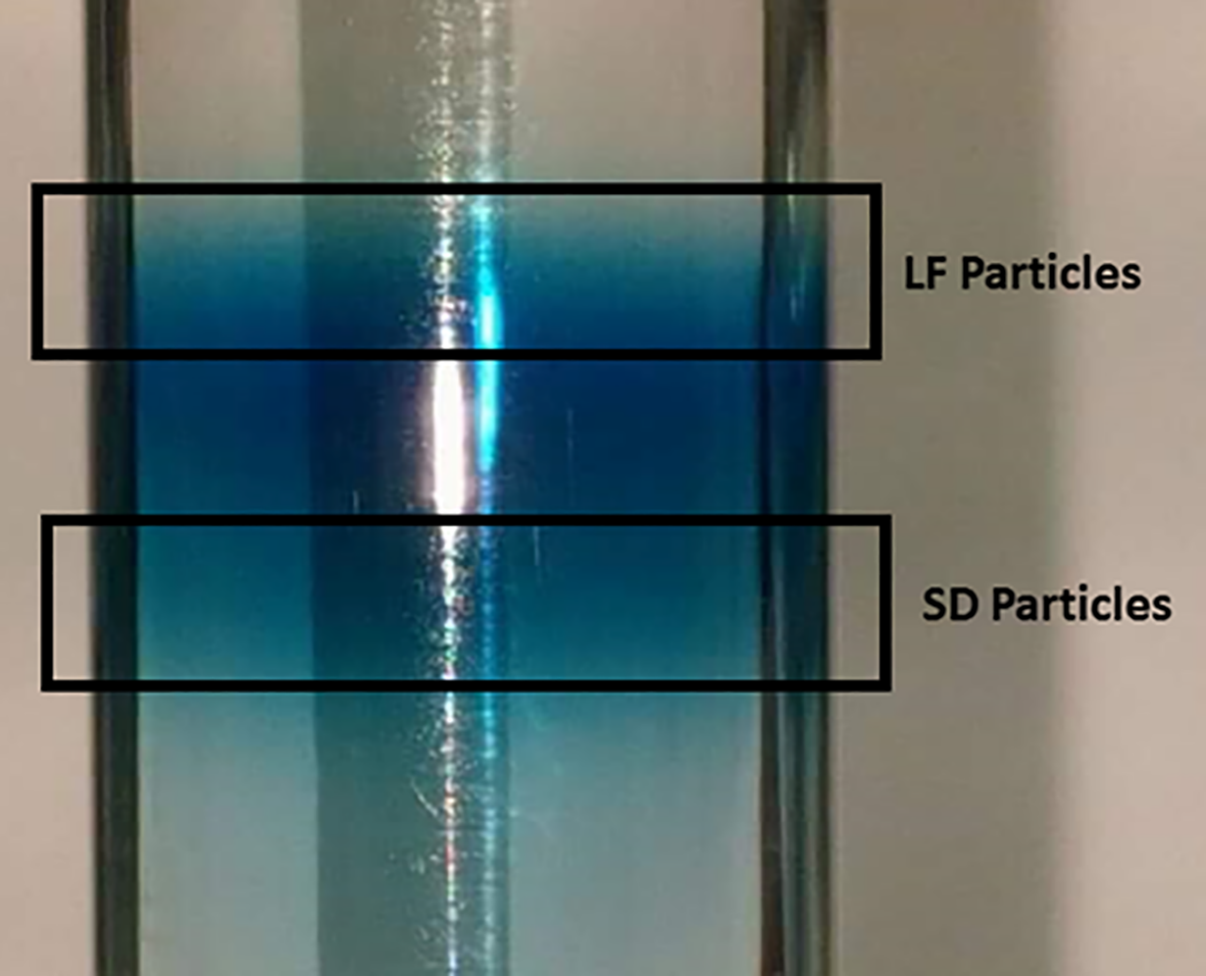
Stained serum bands of large and fluffy (LF) and small and dense (SD) LDL particles.

LDL particles were isolated from normal pooled human serum (Innovative Research, Novi, MI) using an established ultracentrifugation protocol [19]. Briefly, three different density solutions (1.006 g/mL, 1.02 g/mL and 1.065 g/mL) of KBR were prepared to enable the separation of the two layers of LDL (LF- and SD-LDL), which fall at the junction between each layer. The visualization (Fig 1) of the LDL heterogeneity was facilitated by prestaining the serum with Coomassie Brilliant Blue R-250 (Thermo Fisher Scientific, Waltham, MA) prior to density gradient ultracentrifugation using the Beckman Optima XL-100K. The centrifugation was done for 19.5 hrs at 30,600 rpm in 4°C.

### Random library, primers and aptamers

The ssDNA library (TriLink Biotechnologies, San Diego, CA) is a set of 79-mer oligonucleotides composed of a central random region of 40 nt (N40) sandwiched by two primer-binding sites (5’AACTACATGGTATGTGGTGAACT(N40)GACGTACAATGTACCC3’). The primers (Integrated DNA Technologies, Coralville, IA) were the NeoT1 forward primer (5'AACTACATGGTATGTGGTGAACT3') and the NeoT1 Rvs_T7 primer which contains an embedded T7 promoter which was used to transcribe antisense RNA and ultimately isolate ssDNA for further FRELEX selection (5'TAATACGACTCACTATAGGGTACATTGTACGTC3').

After each round of selection, resulting double-stranded DNA PCR product is converted into ssDNA for FRELEX selection. Selected DNA was PCR amplified with the NeoT1 Fwd and NeoT1_T7 Rvs primer. PCR amplification used a 95°C melting temperature, 52°C annealing temperature for the first four cycles, followed by 55°C annealing for all subsequent cycles, and a 72°C extension temperature. After PCR amplification, DNA was purified with the Genejet PCR purification kit (ThermoFisher Scientific, Waltham, MA) and eluted from the column in nuclease free water. The dsDNA with the T7 promoter was transcribed using T7 RNA polymerase (New England Biolabs, Ipswich, MA). The standard protocol for transcription was followed and the reaction was incubated at 37°C for 16 hrs.

The resulting RNA was treated with DNase I (New England Biolabs, Ipswich, MA) for 30 min at 37°C to degrade residual DNA carried over from the PCR reactions. The RNA was then purified using the RNeasy MinElute Cleanup Kit (Qiagen, Hilden, Germany) and eluted from the column with nuclease free water.

The purified RNA is the antisense sequence to the original ssDNA and must be converted back to continue selection. The RNA was reverse transcribed following the standard protocol for Moloney Murine Leukemia Virus (M-MuLV) reverse transcriptase (New England Biolabs, Ipswich, MA). The RNA:DNA hybrid was converted to ssDNA using RNase H (New England Biolabs, Ipswich, MA) and the ssDNA was subsequently purified and carried into the next round of selection.

### FRELEX

**Fig 2:**
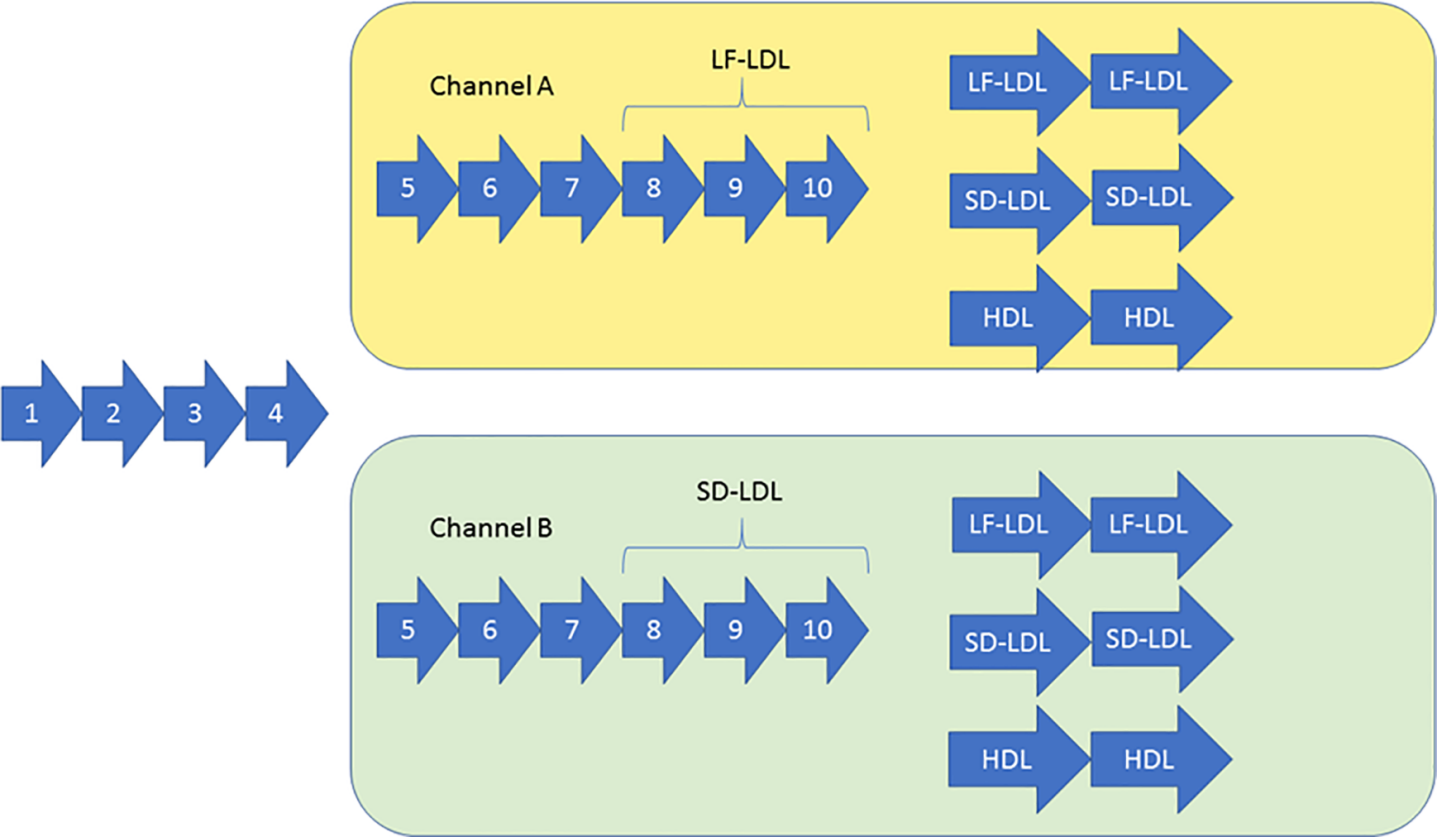
The selection process schematic. LF-LDL, SD-LDL and HDL refer to light and fluffy particles, small and dense LDL particles and HDL particles, respectively.

The FRELEX method developed by NeoVentures Biotechnology Inc. was utilized as previously described. Fig 2 shows the schematic for the specific selection process against Apolipoprotein B100 (ApoB100) on LDL particles. A total of twelve rounds of selection were performed with an increase in selective pressure (decrease in target concentration) across each round. Selection was performed against both targets simultaneously in one channel from selection rounds one to four. Selection was then split into two channels, A and B, from round four and continued for an additional three rounds against both LDL subfraction targets in the separate channels. In selection round eight, selection was directed solely against LF-LDL in Channel A, and SD-LDL in Channel B. After a total of ten rounds of selection, the libraries in each channel were further split into three additional pools against LF-LDL, SD-LDL or HDL. The HDL pool was used as a negative control to later eliminate sequences that were nonselective to LDL. Next generation sequencing (NGS) analysis was performed on selected libraries from selection round eight onwards for both channels.

### Sequence characterization

Nested PCR primers were applied to each selected DNA library from selection rounds 8–12 for sequence identification. The sequences were amplified with these primers, isolated from a 20% acrylamide gel, and purified for sequencing. For purification of the DNA, NGS2 PCR products were run on 20% polyacrylamide gels at 150V for 5 hr. The target band was excised from the gel, fragmented, and stored in TE buffer in a silanized vial for 3 days to elute the DNA. The DNA was subsequently purified using a Genejet PCR purification kit. The DNA was purified following standard protocol and eluted from the column with 30 μl of water. 10 μl of the DNA is run on a 10% polyacrylamide gel to determine concentration of each library. Sequence analysis was completed by the Hospital for Sick Children (Toronto, CA) using Illumina HiSeq 2500.

### Aptamer binding analysis by SPRi

Surface plasmon resonance imaging (SPRi) is a sensitive label-free method used to measure biomolecular interactions through a combination of target flow over a gold chip and the binding of target molecules to aptamers immobilized on the chip. Aptamers for SPRi analysis were synthesized with a disulfide group on the 5’ end and spotted onto a bare gold chip (Xantec Bioanalytics Instrument, Dusseldorf, Germany) in a 1xPBS solution. Thiolated PEG molecules (Creative PEGWorks, North Carolina, USA), applied in two subsequent incubations, were used to block the chip. Gold reduces the disulphide bond on both the aptamers and the PEG and oxidizes the remaining thiols in a strong metallic bond. The result is a corresponding spacer molecule with the blocking group also bound.

The running buffer was composed of 10mM HEPES, 120mM NaCl, 10mM KCl, and 5mM MgCl_2_ at pH 7.4. To remove bound aptamer and target from the chip after measurements, a regeneration solution of 1% SDS followed by 60% DMSO was flowed over the chip. Target molecules were dissolved in PBS and 0.1% Tween buffer to prevent hydrophobic aggregation of particles from artificially amplifying the resonance signal.

Binding analysis was conducted in both buffer and dilute serum. The chip was tested with 1 nM LDL particles in both cases and results were compared to ensure the binding coefficient measurements were reproducible from buffer to serum. Measurements were normalized by subtracting the resonance observed for plain, diluted blood serum from the resonance observed for spiked, diluted blood serum values. Binding to a random DNA sequence was also measured as a negative control and subtracted from the final values.

For analysis, 200μL of target solution was injected into the SPRi (Horiba, Kyoto, Japan) at a flow rate of 10μL/min. This association phase lasted for approximately 20 minutes due to diffusion of the target to the chip surface. The resonance was measured simultaneously in triplicate for each experiment and the average values for each aptamer was computed to calculate the dissociation constant.

Determination of the coefficient of dissociation (k_d_), used the equation:

$$\frac{dx}{dt}=-k_{d}\cdot x$$ (1)

From there, the coefficient of association (k_a_) was computed:

$$\frac{dx}{dt}=\{k_{a}\cdot R_{max}\cdot c-(k_{a}\cdot c+k_{d})\}\cdot x$$ (2)

Both the k_d_ and k_a_ enable the calculation of the individual aptamer k_D_:

$$\frac{k_{d}}{k_{a}}=k_{D}$$ (3)

Here R_max_ is the maximum resonance, c is the concentration of solute being injected, k_a_ is the coefficient of association and k_d_ is the coefficient of dissociation.

Similar procedures were carried out for specificity analysis with HDL particles and HSA in selection buffer. Due to the low resonance values, the injected concentration was increased to 100 nM for HDL and 1 uM for HSA.

### Antisense selection and binding analysis

**Fig 3:**
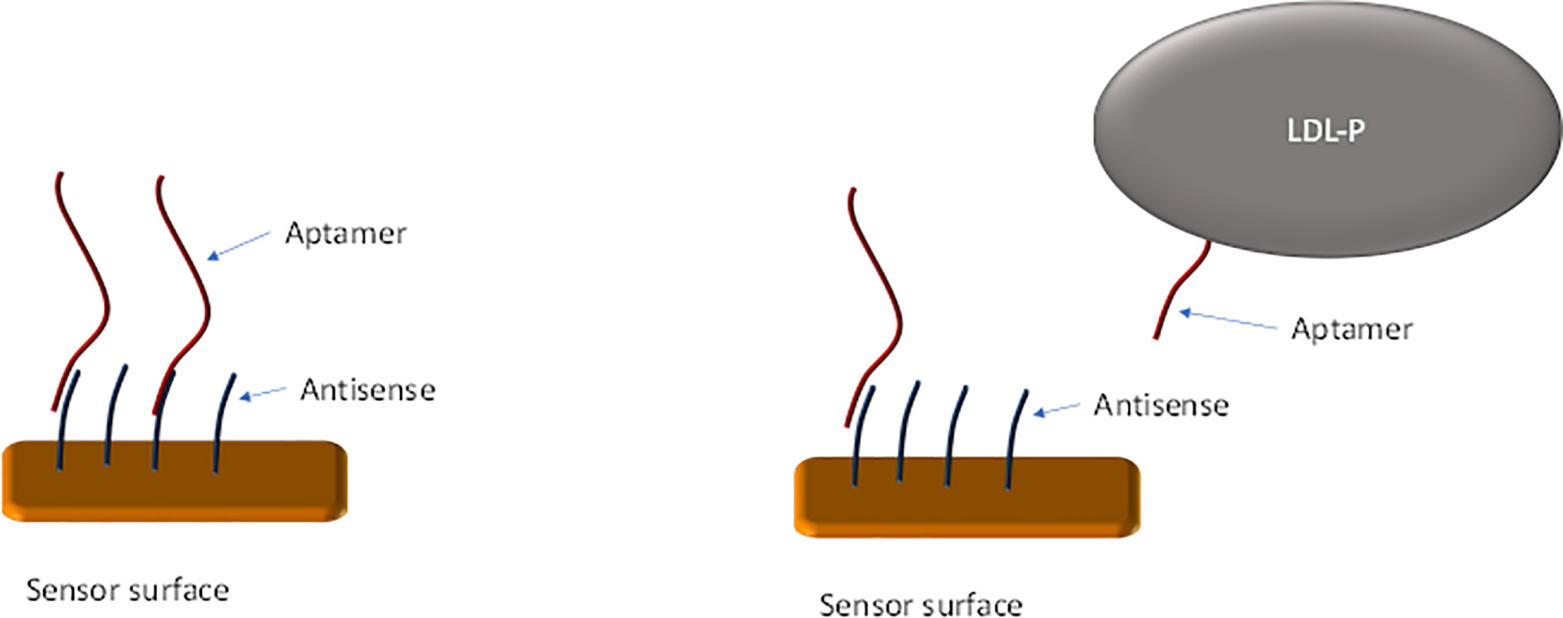
Schematic description of aptamer displacement from antisense strands.

The LDL-P aptamers were developed for use in a variety of diagnostic assay types. One simple and standard diagnostic assay uses aptamers annealed to shorter antisense strands immobilized on a reactive surface as laid out in Fig 3. The aptamers generated in this research were analyzed for potential use in such an assay format.

It is necessary to identify antisense sequences that will bind to the aptamer, but from which the aptamer can still be displaced by interacting with LDL-P. Three antisense strands were designed for the aptamer LDL-A1 and two antisense strands for each of the aptamers LDL-A2 and LDL-A12. Each antisense strand was spotted on a gold chip in triplicate at concentrations of 100 μM and 10 μM. First, the corresponding aptamer was injected at a concentration of 1 μM. Then, without removing the aptamer, commercial LDL particles were injected at a concentration of 100 pM. The resonance curves for each injection were analyzed separately for calculating the dissociation constants.

## Results

### Aptamers selective for LDL particles and subfractions

**Fig 4:**
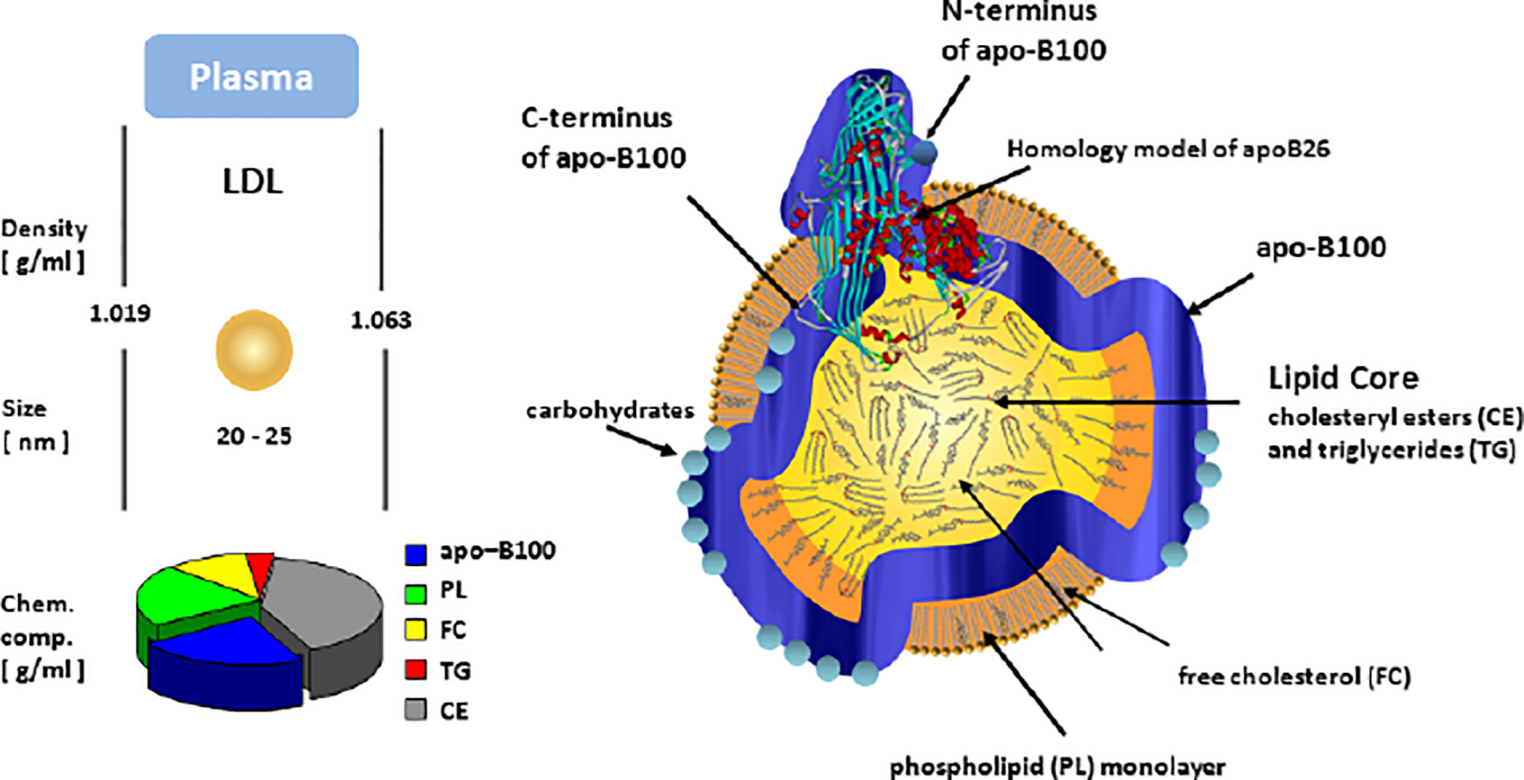
The physical properties of LDL and its surface molecules.

Aptamers that bind to LDL-P and its subfractions were selected using the FRELEX strategy outlined previously. It was hypothesized that aptamers bound to the ApoB100 protein on LDL particles because each LDL particle has one copy of ApoB100 and is otherwise composed of cholesterols and phospholipids as shown in Fig 4. Non-modified nucleotides were used for their lower cost and ease of production with conventional techniques. Furthermore, ssDNA was used preferentially over RNA due to higher stability of DNA molecules in serum.

The initial goal of this project was to identify aptamers that bind selectively to LDL particles with the secondary goal of selecting aptamers that can discriminate between LF-LDL and SD-LDL particles.

Four to nine million sequences were captured after each selection round for a total of approximately 100 million sequences across twelve selection rounds. To normalize for variation in the number of sequences observed after each round, the number of copies of each sequence is divided by the total number of sequences collected from that round. This value, known as the sequence frequency, is used to name sequences. For example, LDL-A1 is the sequence that appears with the highest percentage at the end of selection round 10 in Channel A.

The top twenty sequences by sequence frequency at the end of selection round 10 were categorized as preliminary candidates. The change in sequence frequency across the last three rounds of selection was graphed for each preliminary candidate. A more positively sloped trajectory implies more rapid positive enrichment and potential for a strong aptamer. For Channel A, sequence LDL-A1 dominated selection with 280,300 copies observed in selection round 10. LDL-A12 exhibited an above-average enrichment rate and demonstrated the largest difference in enrichment between the LF-LDL and SD-LDL selection channels. This higher rate of enrichment may be an indication that LDL-A12 favors binding LF-LDL. Results for Channel B were comparable to Channel A.

**Fig 5:**
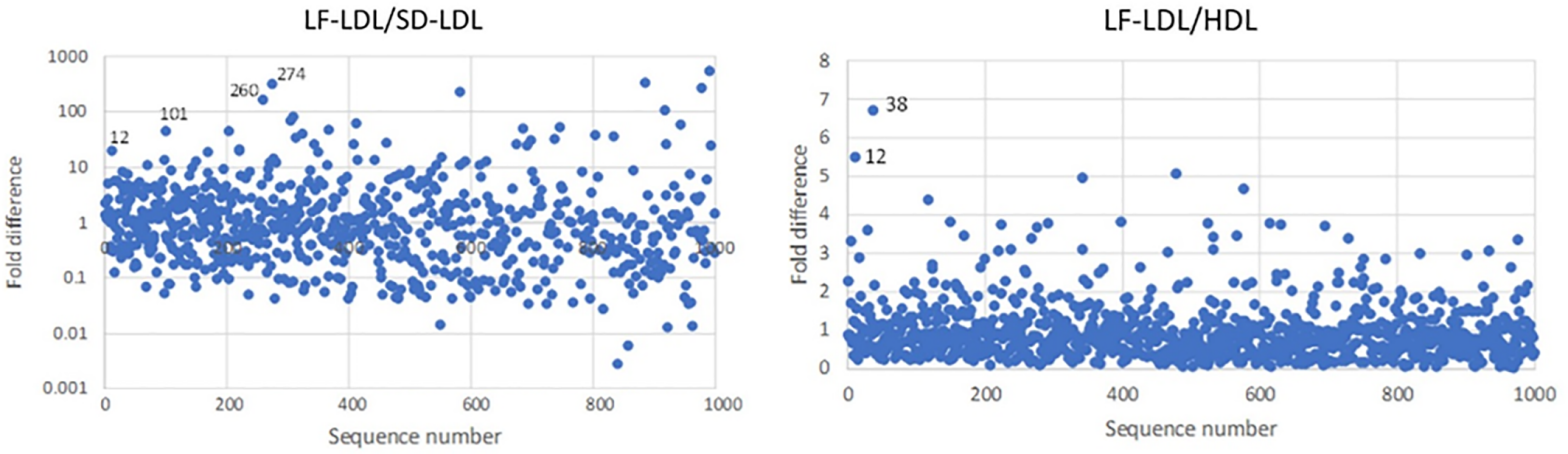
Specificity of enrichment towards LF-LDL in the top 1000 sequences in Channel A. The top 1000 sequences by sequence frequency at the end of selection round 10 in Channel A were analyzed for specificity. (A) The ratio of sequence frequency between the LF-LDL and SD-LDL pools after two further rounds of selection showed A12, A101, A260 and A274 to be potential candidates for further binding studies. (B) The ratio of sequence frequency between the LF-LDL and HDL pools after two further rounds of selection showed A12 and A38 to be potential candidates for further binding studies. The overlap of A12 suggests a promising candidate.

In each channel, the final two rounds of selection after round 10 saw the library split equally into three pools to focus on identifying sequences with specific affinity to the targeted LDL particles and eliminate sequences that demonstrated non-specific enrichment. In Channel A, selection was focused on identifying sequences with greater affinity for LF-LDL than SD-LDL and HDL. Analysis of the data shown in Fig 5 indicated the sequences LDL-A1, LDL-A2, LDL-A12, LDL-A38, LDL-A260 and LDL-A274 to be candidate sequences in binding assays. LDL-A12 showed overlapping selectivity for LF-LDL over SD-LDL and HDL, confirming potential for quantifying subfractions.

**Fig 6:**
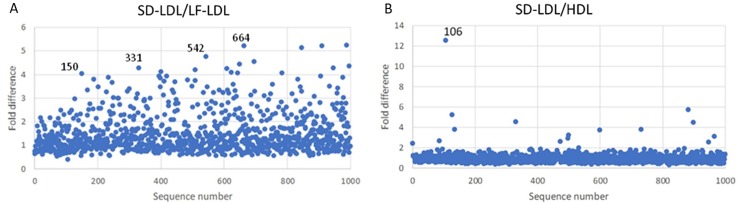
Specificity of enrichment towards SD-LDL in the top 1000 sequences in Channel B. The top 1000 sequences by sequence frequency at the end of selection round 10 in Channel B were analyzed for specificity. (A) The ratio of sequence frequency between the SD-LDL and LF-LDL pools after two further rounds of selection showed B150, B331, B542 and B664 to be potential candidates for further binding studies. (B) The ratio of sequence frequency between the SD-LDL and HDL pools after two further rounds of selection showed B106 to be a potential candidate for further binding studies. No overlapping sequences were found.

In Channel B, sequences with selective preference for SD-LDL over LF-LDL and HDL were identified. As shown in Fig 6, though several sequences exhibited preferential binding to SD-LDL, there was no overlap of sequences between those that bound to SD-LDL over LF-LDL and those that bound to SD-LDL over HDL. This limits the ability to selectively quantify SD-LDL subfraction. Sequences B150, B331, B542 and B664 were added as candidates for binding analysis as they demonstrated elevated levels of enrichment for SD-LDL over LF-LDL. Sequence LDL-B106 was also added as it exhibited the strongest difference between SD-LDL and HDL.

### Aptamers bind commercial LDL particles with high affinity and specificity

**Fig 7:**
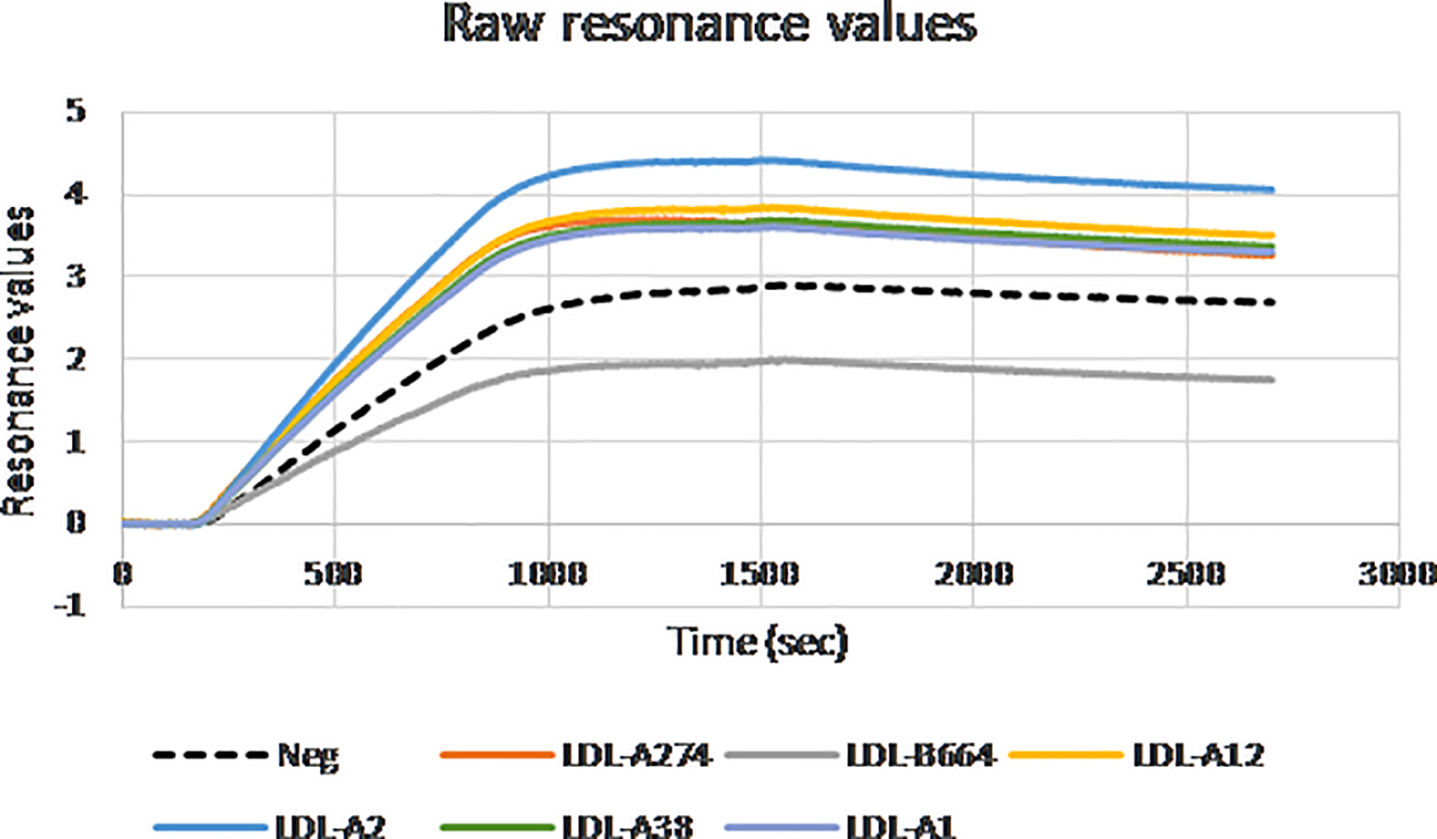
Raw resonance curves from SPRi binding analysis for selected aptamer sequences.

The following final sequences were selected for SPRi binding analysis based on sequence frequency, rate of enrichment and selectivity towards LDL particles and their subfractions: LDL-A1, LDL-A2, LDL-A12, LDL-A38, LDL-A274, LDL-B664 and a negative control (random sequence). As shown in Fig 7, the negative aptamer exhibited a higher resonance than the aptamer LDL-B664 but lower resonance than the other aptamers, eliminating LDL-B664 from further analysis. This was not unexpected as LDL-B664 had a final sequence frequency that was ranked 664^th^ in Channel B.

**Fig 8:**
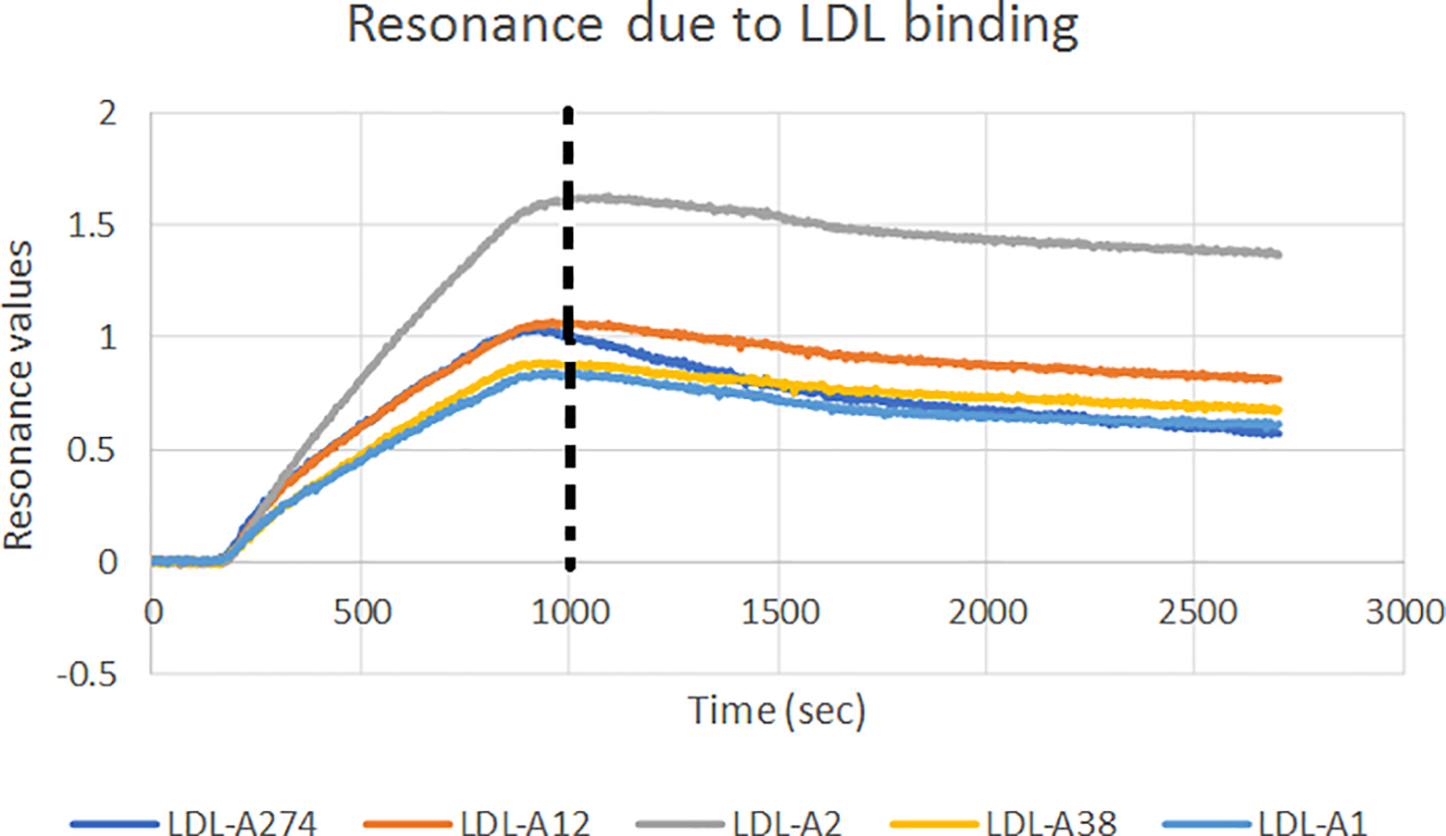
Decrease in resonance after the dotted line conveys the dissociation rate of the complex.

The vertical dashed line in Fig 8 refers to the differentiation point between association and disassociation. Prior to this line, LDL particles flow over the aptamers at a steady concentration of 1 nM. After this line, LDL particles are no longer flowing over the aptamers, and the dissociation rate of the LDL-aptamer complex can be measured. The full set of aptamers were tested in triplicate twice to confirm the results.

The absence of binding data for certain aptamers in Table 1 does not mean those aptamers did not bind to the target. In some cases, an upward tail was observed in the disassociation phase preventing us from determining the binding coefficients. This was likely due to high affinity causing the aggregation of LDL particles. To control for aggregation, the experiments were repeated in 0.1% Tween which did not influence the results. Calculated aptamer binding coefficients using Eqs 1, 2 and 3 are displayed in Table 1.

**Table 1:** Coefficients of dissociation (k_d_), association (k_a_) and binding (k_D_) in buffer.

Aptamer	Type of LDL	k_d_ (M^2^)	k_a_ (M)	k_D_ (M)
LDL-A1	All LDL-P	3.69E-04	6.50E+06	5.31E-11
	SD-LDL	1.45E-03	4.80E+06	3.02E-10
	LF-LDL	2.36E-04	7.23E+06	3.27E-11
LDL-A2	All LDL-P	2.61E-04	6.15E+06	4.21E-11
	SD-LDL	-	-	-
	LF-LDL	1.79E-04	1.13E+07	1.59E-11
LDL-A12	All LDL-P	3.35E-04	6.58E+06	4.97E-11
	SD-LDL	1.14E-03	4.95E+07	2.30E-11
	LF-LDL	-	-	-
LDL-A38	All LDL-P	3.00E-04	6.50E+06	4.55E-11
	SD-LDL	2.34E-03	9.99E+06	2.34E-10
	LF-LDL	-	-	-
LDL-A274	All LDL-P	6.48E-04	7.55E+06	7.73E-11
	SD-LDL	1.26E-03	9.29E+06	1.36E-10
	LF-LDL	8.80E-04	2.83E+06	4.82E-10

Based on the data, LDL-A1 and LDL-A38 appear to have some promise for selectively binding LF-LDL over SD-LDL. The remaining aptamers show similar affinities to both subfractions.

### Aptamers bind LDL-P with high affinity and specificity in human serum

In order to utilize these aptamers in a diagnostic assay, they must bind specifically in human serum. To study this, SPRi measurements were conducted in buffer again, to verify consistent results, and then in 1% blood serum. This binding data is shown in Table 2.

**Table 2:** Coefficients of dissociation (k_d_), association (k_a_) and binding (k_D_) in 1% serum.

Aptamer	Sample	k_d_ (M^2^)	k_a_ (M)	k_D_ (M)
LDL-A1	Buffer	1.17E-04	5.47E+06	2.14E-11
	Serum	-	-	-
LDL-A2	Buffer	8.99E-05	5.98E+06	1.5E-11
	Serum	5.21E-05	1.02E+07	5.12E-12
LDL-A12	Buffer	7.54E-05	7.01E+06	1.08E-11
	Serum	4.15E-04	8.90E+06	4.66E-11
LDL-A38	Buffer	1.17E-04	5.65E+06	2.07E-11
	Serum	9.09E-05	5.10E+06	1.78E-11
LDL-A274	Buffer	1.17E-04	5.47E+06	2.14E-11
	Serum	5.89E-04	5.17E+06	1.14E-10

For LDL-A1, the k_D_ could not be measured in blood serum because the disassociation curve did not curve downwards. Therefore, it can be assumed that the k_D_ value for this aptamer was not affected by the presence of blood serum. Aptamers LDL-A274 and LDL-A12 both displayed lower affinities in blood serum, while aptamers LDL-A2 and LDL-A38 appeared to be unaffected.

Finally, aptamer selectivity was tested with HDL and HSA in buffer. As expected, the HDL signal and corresponding binding affinities were much lower than for LDL-P as shown in Table 3.

**Table 3:** The binding coefficients for selected aptamers to HDL in buffer.

Aptamer	Target	k_d_ (M^2^)	k_a_ (M)	k_D_ (M)
LDL-A1	HDL	4.01E-04	4.86E+04	8.26E-09
LDL-A2	HDL	4.18E-04	5.77E+04	7.25E-09
LDL-A12	HDL	5.67E-04	5.85E+04	9.69E-09
LDL-A38	HDL	5.92E-04	5.14E+04	1.15E-08
LDL-A274	HDL	6.56E-04	7.28E+04	9.01E-09

**Fig 9:**
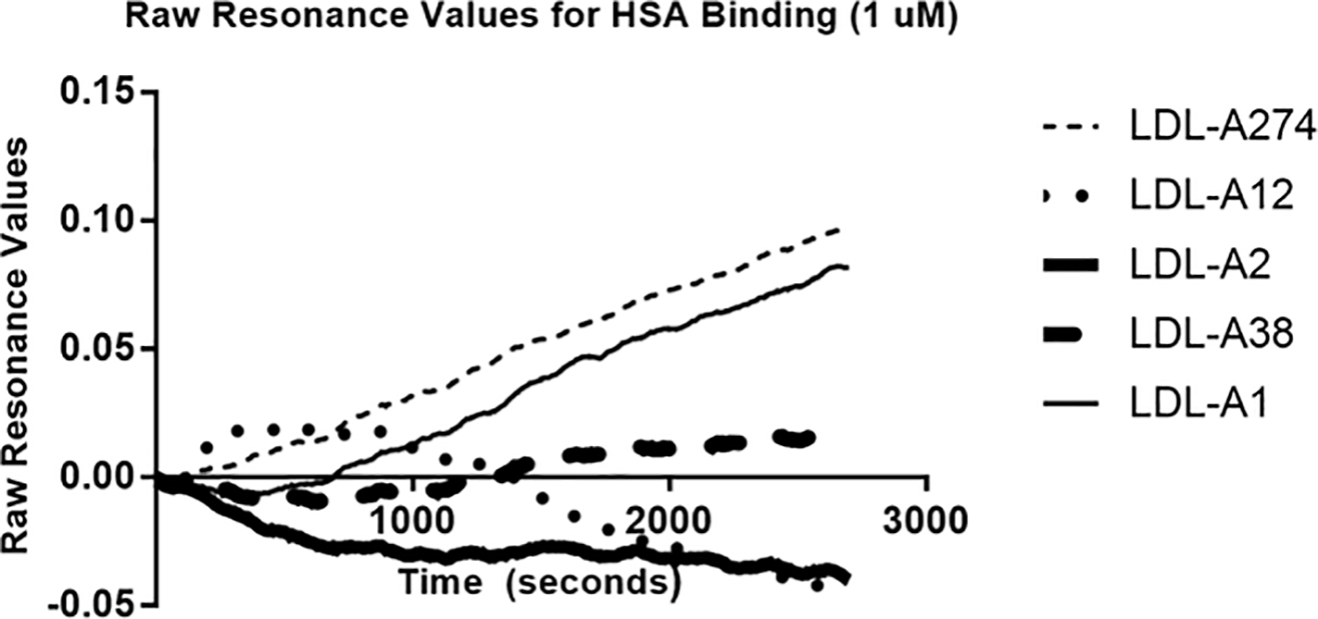
The resonance values over time of the aptamers binding to human serum albumin (HSA) in selection buffer.

LDL-A1 exhibited a binding affinity difference of slightly over 5,000-fold for LDL-P versus HDL; however, the rest of the aptamers exhibited binding affinity differences of around 500-fold for LDL-P versus HDL. In all cases, the selected aptamers demonstrated strong selectivity towards LDL-P over HDL. Furthermore, for HSA binding (Fig 9), the resonance values were near zero and the curves did not flatten to indicate aptamer binding despite the injection of 1 uM HSA. This further confirmed the selected aptamers’ utility in a diagnostic assay.

### Selection of antisense sequences for use in a diagnostic assay

The LDL-P aptamers developed are intended for application in an electrochemical-based diagnostic system. One simple and standard diagnostic assay uses the aptamers annealed to shorter antisense strands immobilized on a reactive surface as laid out in Fig 3. Aptamers dissociating from the antisense strands upon binding to LDL-P can be measured by tagging the aptamer with an electrochemically active molecule (e.g. methylene blue, ferrocene) that decreases in electrochemical activity upon dissociation. To achieve this, it is necessary to identify antisense sequences that will bind to the aptamer, but from which the aptamer is still displaceable by LDL-P. Antisense strands were designed for the aptamers with the highest affinities to LDL-P. Three antisense strands were tested for the aptamer LDL-A1 and two antisense strands for each of the aptamers LDL-A2 and LDL-A12 as shown in Table 4.

**Table 4:** The different antisense strands and associated sequences.

Strand	Sequence
LDL-A1.1	TAAAATCGAGGTAG
LDL-A1.2	GTTGGTAAGTGAAATA
LDL-A1.3	GTTGGTAAGCGCGAGG
LDL-A2.1	CTGACAGTTCACCAT
LDL-A2.2	GACGAGAGCCAAGGTGAA
LDL-A12.1	ACAAGGGTAAATAAGGT
LDL-A12.2	GATGGAAATGATAAAG

**Fig 10:**
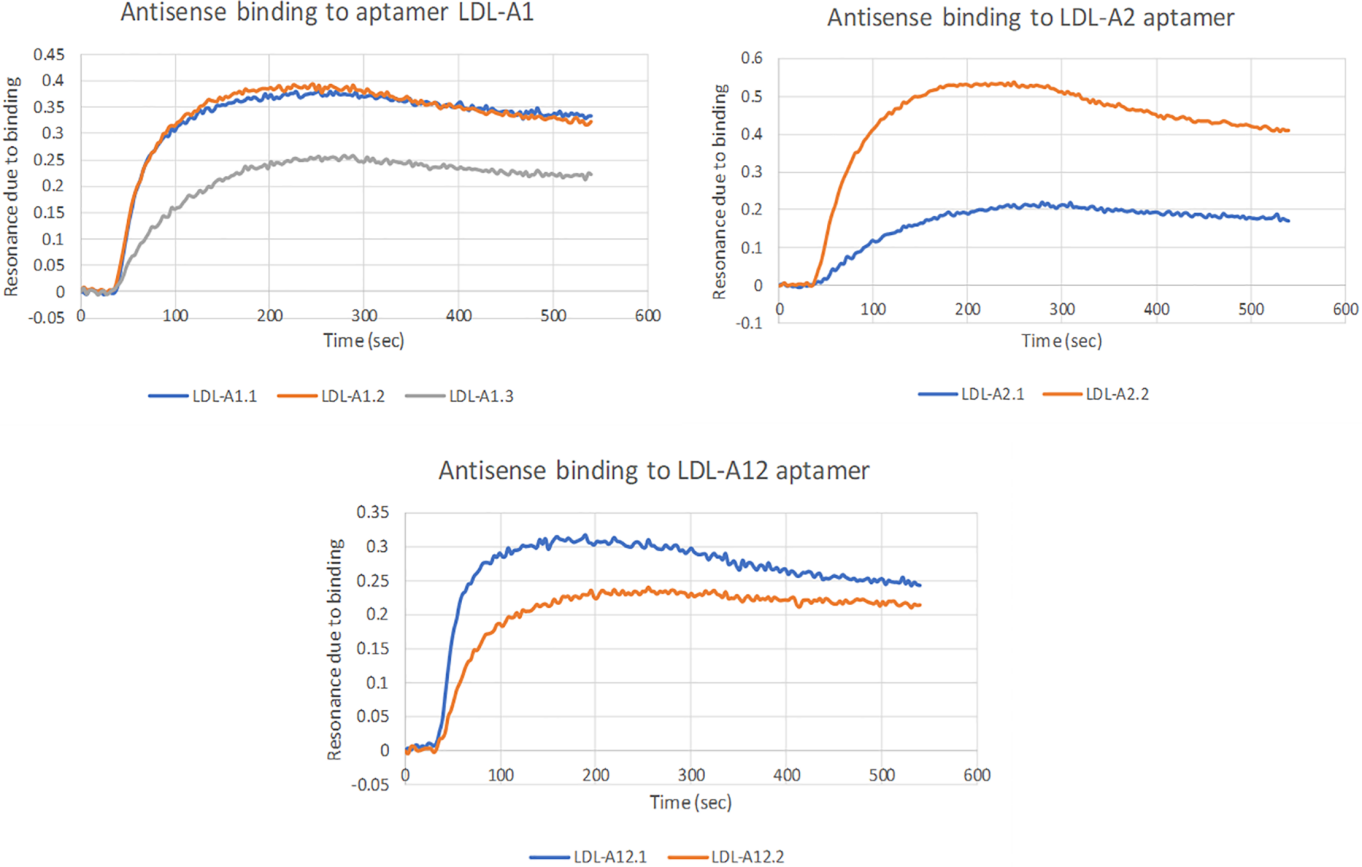
Resonance curves of antisense binding for LDL-A1, LDL-A2 and LDL-A12.

The method for resonance testing previously outlined was repeated here. Briefly, antisense strands were spotted in triplicate on gold chips and respective aptamers were injected at a concentration of 100 μM. Negative controls were performed for each aptamer using a chip spotted with three random sequences. The binding curves in Fig 10 were determined by subtracting the average resonance of the three random strands from the average resonance for each antisense strand.

**Fig 11:**
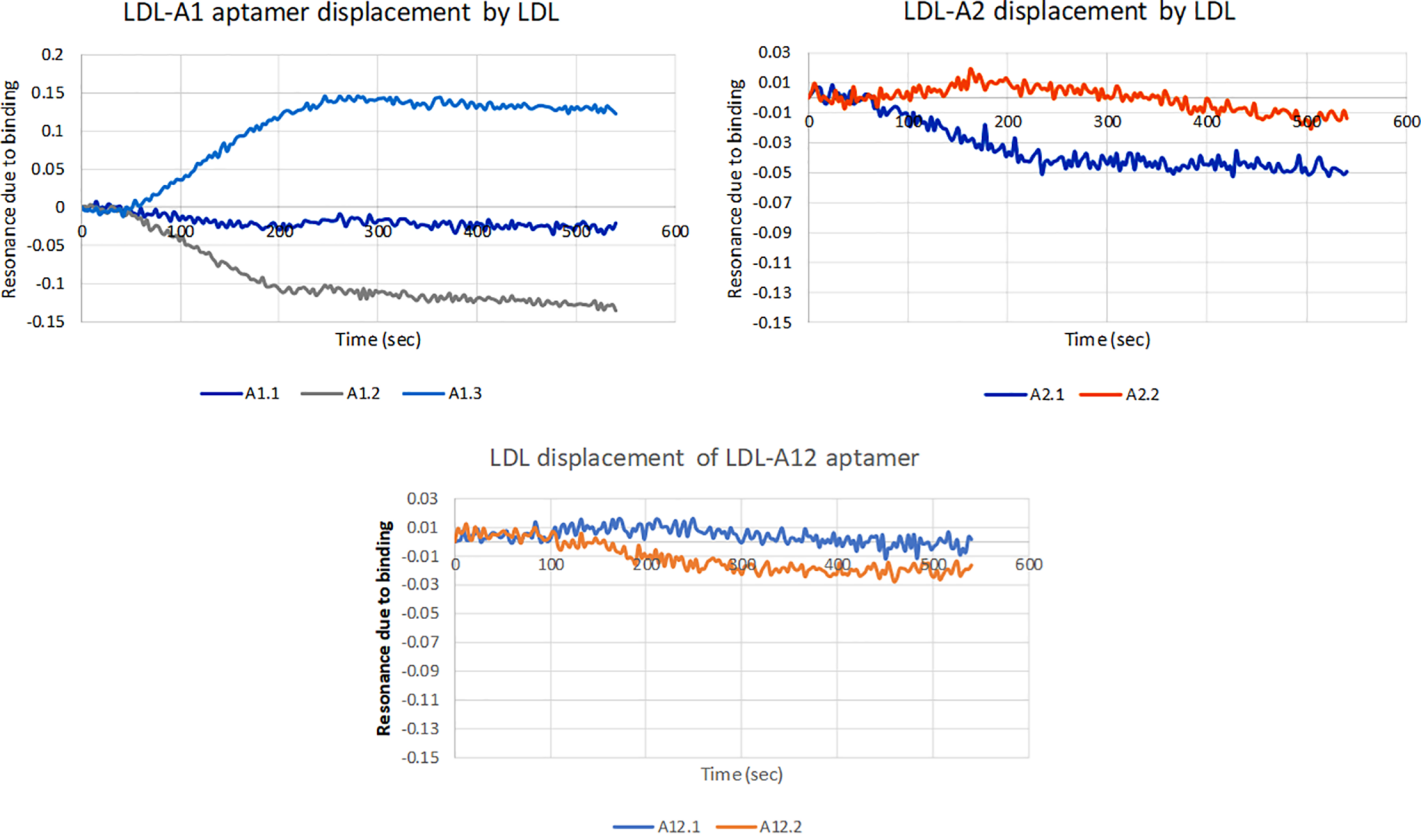
Resonance curves for the displacement of LDL-A1, LDL-A2 and LDL-A12 by LDL.

Once aptamer-antisense complexes were formed, LDL-P was added and a decrease in resonance was measured as the aptamers interacted with LDL-P and disassociated from the antisense strands. The resulting resonance decrease was more significant for antisense strands with lower molecular weights. The LDL-A1.3 antisense strand was an exception to this pattern. Fig 11 shows the resulting averaged resonance curves with the curves from Fig 10 subtracted to show net change.

The antisense strand LDL-A1.3 exhibited an increase in resonance even after subtraction meaning either the aptamer was able to bind to LDL-P while hybridized to the antisense or the antisense itself exhibited some capacity to bind to LDL-P. LDL-A1.1 exhibited very weak displacement, while LDL-A1.2 exhibited strong displacement. Though the aptamer/antisense binding was similar between these two antisense, the LDL displacement is different.

For the LDL-A2 aptamer, displacement was observed from the A2.1 antisense but not for A2.2. This was expected as the LDL-A2.1 antisense hybridized less strongly with this aptamer than the LDL-A2.2 antisense. For LDL-A12, neither of the antisense were displaced effectively by LDL. Based on this data set, the best design for a diagnostic assay based on aptamer displacement would use the LDL-A1 aptamer with the LDL-A1.2 antisense.

## Discussion

CVD remains the leading cause of death worldwide. Nevertheless, CVD risk assessment for patients remains suboptimal with the urgent need for simpler, faster and lower-cost methods for advanced lipoprotein testing. Aptamers serve as a promising solution for use in a diagnostic assay format that improves upon the current testing paradigm. In this research, aptamers were generated as ligands that can bind LDL particles. Furthermore, an agnostic selection for potential epitopic differences between types of LDL particles was used to potentially enable subfraction quantification. This application is not feasible with antibodies due to their rigidity and size. The selection of ssDNA aptamers presumably against the ApoB100 protein on LDL particles allowed for the isolation of five aptamers: LDL-A1, LDL-A2, LDL-A12, LDL-A38, LDL-A274—that bind to LDL particles selectively and with high affinities.

SPRi binding analysis revealed higher than expected binding affinities with all five of the aptamers having k_D_ values in the low picomolar range. Initially, the two hypotheses for the high binding affinities were either that the aptamers may be recognizing more than one site per LDL particle or the system was detecting aggregation of LDL particles on the chip—both of which would lead to signal amplification. To control for aggregation, further testing was done using 0.1% Tween buffer, a nonionic surfactant. This buffer solution was spiked with both commercial LDL and mixed in serum samples. The signal remained comparably high, weakening the case for aggregation being the reason for the high affinity measurements. This implies that the aptamers may be targeting multiple epitopes on ApoB100 or epitopes entirely independent of ApoB100 on LDL particles which would be useful in a sandwich assay format.

It is encouraging to observe the high affinities and specificities of the five selected aptamers to LDL-P. Additionally, there were differential affinities between the aptamers for different LDL subfractions. Numerous studies have demonstrated the clinical significance of LDL subfractions for further risk stratification beyond LDL-P measurement. LDL-A1 and LDL-A38 had approximately 10x higher affinity for LF-LDL particles compared to SD-LDL, demonstrating the possibility using aptamers for selective LDL-P subfraction quantification. All of the selected aptamers demonstrated picomolar range affinities in both buffer and dilute serum as shown in Table 2 and high specificity to LDL over HDL and HSA, making them promising candidates for use in a diagnostic assay.

Electrochemical detection is an excellent assay format to enable simple, rapid measurement of LDL-P in clinical matrices. One such assay format was explored herein. Aptamers were annealed to shorter antisense strands that are immobilized on a reactive surface and then dissociated from the antisense strands upon binding to LDL-P. This dissociation can be paired with a change in electrochemical signal by attaching a redox label (e.g. methylene blue) to the aptamer. Antisense strands were developed and tested for enabling this detection format. Three antisense constructs for LDL-A1 and two antisense constructs for LDL-A2 and LDL-A12 were tested, resulting in a promising candidate for future use in this assay format. By having high affinity aptamers, significant dilution of the clinical sample should also be feasible. This allows for the minimization of matrix effects and interfering substances in serum while still enabling sensitive measurement in the clinical range.

Numerous antibodies have been developed previously for quantifying LDL cholesterol. However, to our knowledge, this is the first study to report the binding of aptamers to LDL particles with high affinity and selectivity. Furthermore, our results demonstrate that it may be possible to use the identified aptamers to selectively quantifying subfractions of LDL-P based on particle size and density. Overall, our results show that the aptamers described here, and their corresponding antisense strands, demonstrate sufficient affinity and specificity for use in a simple, clinically relevant diagnostic assay for measuring LDL-P in a low-cost, rapid manner.

## Supporting information

S1 Dataset Experimental descriptions and raw SPRi data. (ZIP)
